# Space-time variation of malaria incidence in Yunnan province, China

**DOI:** 10.1186/1475-2875-8-180

**Published:** 2009-07-31

**Authors:** Archie CA Clements, Adrian G Barnett, Zhang Wei Cheng, Robert W Snow, Hom Ning Zhou

**Affiliations:** 1 University of Queensland, School of Population Health, Herston, Queensland, Australia; 2 Australian Centre for International and Tropical Health, Queensland Institute of Medical Research, Herston, Queensland, Australia; 3 Institute of Health and Biomedical Innovation, Queensland University of Technology, Kelvin Grove, Queensland, Australia; 4 Yunnan Institute of Parasitic Diseases, Pu'er, Yunnan, PR China; 5 Malaria Public Health and Epidemiology Group, Centre for Geographic Medicine, KEMRI – University of Oxford – Wellcome Trust Collaborative Programme, Nairobi, Kenya; 6 Centre for Tropical Medicine, Nuffield Department of Clinical Medicine, University of Oxford, CCVTM, Oxford, UK

## Abstract

**Background:**

Understanding spatio-temporal variation in malaria incidence provides a basis for effective disease control planning and monitoring.

**Methods:**

Monthly surveillance data between 1991 and 2006 for *Plasmodium vivax *and *Plasmodium falciparum *malaria across 128 counties were assembled for Yunnan, a province of China with one of the highest burdens of malaria. County-level Bayesian Poisson regression models of incidence were constructed, with effects for rainfall, maximum temperature and temporal trend. The model also allowed for spatial variation in county-level incidence and temporal trend, and dependence between incidence in June–September and the preceding January–February.

**Results:**

Models revealed strong associations between malaria incidence and both rainfall and maximum temperature. There was a significant association between incidence in June–September and the preceding January–February. Raw standardised morbidity ratios showed a high incidence in some counties bordering Myanmar, Laos and Vietnam, and counties in the Red River valley. Clusters of counties in south-western and northern Yunnan were identified that had high incidence not explained by climate. The overall trend in incidence decreased, but there was significant variation between counties.

**Conclusion:**

Dependence between incidence in summer and the preceding January–February suggests a role of intrinsic host-pathogen dynamics. Incidence during the summer peak might be predictable based on incidence in January–February, facilitating malaria control planning, scaled months in advance to the magnitude of the summer malaria burden. Heterogeneities in county-level temporal trends suggest that reductions in the burden of malaria have been unevenly distributed throughout the province.

## Background

Despite significant reductions in the overall burden of malaria in the 20^th ^century, this disease still represents a significant public health problem in China [1], with > 50,000 confirmed and > 80,000 suspected cases and 18 deaths reported in 2007 [2]. The south-western province of Yunnan has one of the highest malaria burdens in China. This province has international borders with Laos, Myanmar and Vietnam and impedance of effective malaria control and repeated epidemics have been associated with social and economic migration of people across these borders [3,4].

As with other vector-borne diseases, malaria typically shows temporal and spatial variations that are driven by climatic, ecological and human factors [5-9]. These correlates can be used to predict the spatio-temporal distribution of disease burden and thus guide the cost-effective allocation of intervention resources in space and time. For malaria, there has been considerable interest in the development of malaria early warning systems (MEWS) based on aberrant values in time series surveillance data, climate signals (potentially derived from remote sensing data [10,11]), or a combination of the two [12], to enable interventions to be instigated well before the epidemics take hold [13,14]. Examples include use of surveillance data to predict epidemics in Ethiopia [15], rainfall data to predict epidemics in Kenya [16] and the El Niño Southern Oscillation index to predict epidemics in Kenya [17] and Southern Asia [18]. In the current study, spatial, intra-annual and inter-epidemic patterns of malaria in Yunnan were explored using a Bayesian analysis of time-series data on reported *Plasmodium falciparum *and *Plasmodium vivax *clinical events over 16 years.

## Methods

### Study area

**Figure 1:**
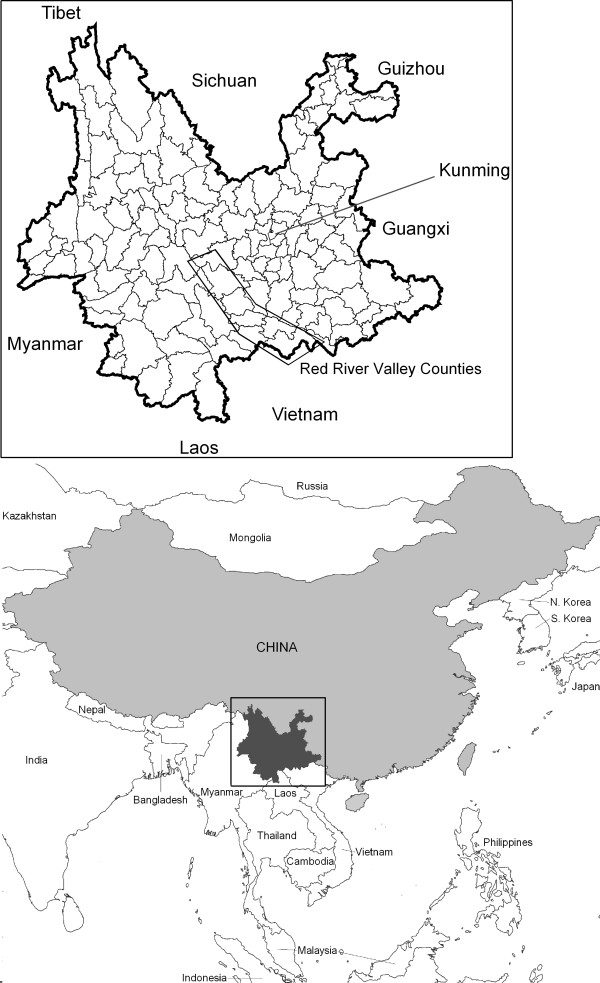
**Features of Yunnan Province relative to neighbouring provinces and countries**.

Yunnan province (Figure 1) has an area of 394,000 square kilometres and a population during the 2000 census of 42.9 million. The province is divided into 16 prefectures, 128 counties, and over 1,400 townships. The climate is characterized by a cool, dry winter and a warm, wet summer, with peaks in rainfall in July–August and temperature in May–August. Elevation is highly variable, with county median elevation ranging from just over 600 metres above sea level in the south-east (Hekou county) to over 3,600 metres in the north-west, close to Tibet (Deqin county). See the additional file 1 for more information on the climate and geography of Yunnan.

### Malaria surveillance data

The Chinese Centre for Disease Control lists malaria as a notifiable disease and maintains routine surveillance of clinical malaria nationwide. Diagnosis of malaria is based on clinical signs and, in an unspecified proportion of cases, laboratory confirmation. Cases of malaria diagnosed at health facilities are reported to the township-level authorities, and collated up through the county, prefecture and provincial administrative levels. For the purposes of the present study, reported numbers of *P. vivax *and *P. falciparum *malaria cases were assembled by month for each of the 128 counties between January 1991 and December 2006.

### Climate, elevation and population data

High-resolution (1 square kilometre) raster maps of interpolated long-term average monthly rainfall and minimum and maximum temperature were obtained from the *WORLDCLIM *website [19,20]. Digital elevation (in meters above sea level for cells of a 1 square kilometre grid), were also obtained from the same source. Rainfall, temperature and elevation maps were imported into a geographical information system (GIS; ArcView version 9, ESRI, Redlands, CA) and linked spatially to a digitized boundary map of the 128 counties across Yunnan. The county median values of rainfall, temperature and elevation were computed in the GIS to define attribute parameters in subsequent models. Data from national censuses conducted in 1990 and 2000 were used to impute inter-censual growth rates at the county level and to estimate population counts for each month between January 1991 and December 2006.

### Statistical analysis

An initial descriptive analysis of malaria incidence was conducted. Crude standardized morbidity ratios (SMRs) for each county were calculated by dividing the observed number of cases across 1991–2006 by the expected number, where the expected was calculated by multiplying the provincial incidence by the average population for each county across 1991–2006. Pairwise Pearson cross-correlations for each month (January–December) were calculated to investigate temporal correlation in the case incidence data. Due to the strong correlations between rainfall and minimum temperature (Pearson's correlation = 0.83) and minimum and maximum temperature (0.89), only rainfall and maximum temperature were included in subsequent models. Poisson regression suggested that elevation was not significantly associated with *P. vivax *or *P. falciparum *case incidence after accounting for the effects of rainfall and temperature and elevation was thus excluded from the final models.

Bayesian spatiotemporal Poisson regression models were constructed using the WinBUGS software, version 1.4.3 (MRC Biostatistics Unit, Cambridge, UK). The Bayesian approach is advantageous in that it provides a convenient platform for modelling hierarchical datasets and incorporating spatiotemporal autocorrelation. Similar Bayesian spatiotemporal models of malaria surveillance data have been reported previously [21,22]. The outcome was the monthly number of cases of *P. vivax *or *P. falciparum *malaria in each county and the offset was the expected number of cases based on the population of each county for the months January 1991 to December 2006.

Fixed effects were added for monthly rainfall and maximum temperature and random effects were added for inter-annual variation in incidence in January–February and June–September, with incidence in January–February modelled (using a linear regression) as a predictor of incidence in June–September (this was justified by strong correlations of raw incidence in the summer with raw incidence in the preceding January and February). Additional components included a spatial county-level random effect, modelled using a conditional autoregressive (CAR) prior structure [23] and fixed-effect temporal trends (i.e., where the number of months passed since the index month, January 1991, was regressed against the outcome) with: 1) an overall provincial mean trend and 2) spatially smoothed county-level trends, where spatial variation was modelled by fitting a CAR prior to the trend coefficients as per Bernardinelli and Montomoli [24]. Statistical notation of the final model is provided in additional file 2.

Three chains of the model were run consecutively, with each model parameter monitored after an initial burn-in of 1,000 iterations. Convergence was assessed by visual inspection of history and density plots of the three chains. Convergence occurred within the first 10,000 iterations for each model, but some parameters were slow-mixing, with considerable autocorrelation. Therefore, subsequent iterations were thinned by a factor of 10. Ten thousand values from the posterior distribution of each parameter were stored and summarized using descriptive statistics (posterior mean, 95% posterior credible interval [CrI]). Choropleth maps of model outputs were created in the GIS.

## Results

### Descriptive analysis

**Figure 2:**
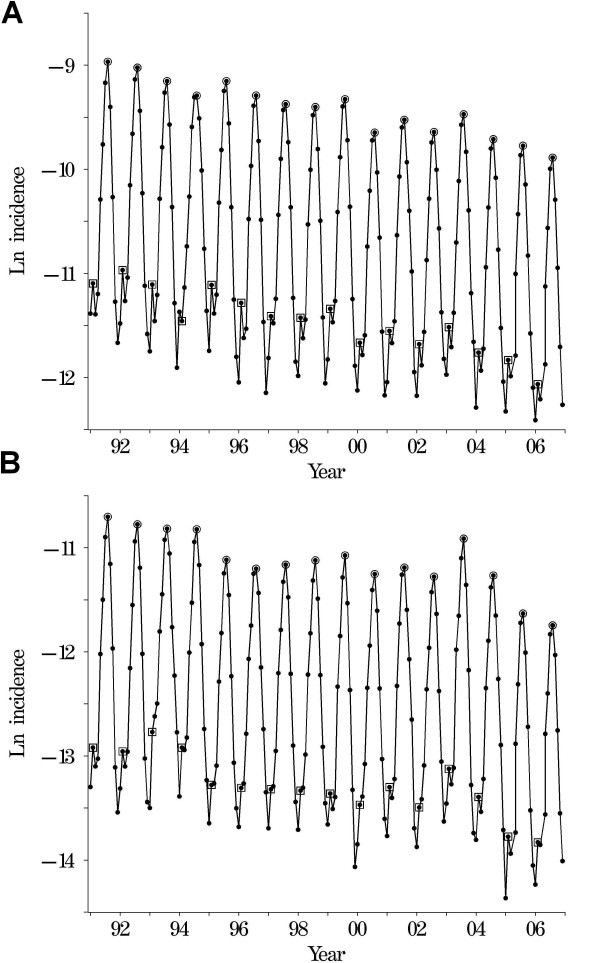
**Incidence of a) *Plasmodium vivax *and b) *P. falciparum *malaria in Yunnan province, January 1991–December 2006**. The squares indicate February and the circles August.

In Yunnan, there was a total of 250,070 cases of *P. vivax *malaria documented over the 16 years of observation (1991–2006), representing a crude incidence of 3.12 cases per 100,000 person years at risk. There were 44,465 cases of *P. falciparum *malaria reported during the same period representing a crude incidence of 0.55 cases per 100,000 person years at risk. Across the entire time-series, 10 counties had no reported cases of *P. vivax *and 29 counties had no reported cases of *P. falciparum*. The monthly incidence for both *P. vivax *and *P. falciparum *is shown in Figure 2 (note the natural log scale of the *y*-axis, used to help visualisation of temporal variation). Some features are immediately apparent for both types of malaria: there was a strong seasonal pattern characterized by a peak of malaria incidence in summer (June–September, peak month August) and a trough in winter (November–January). A smaller peak preceding the summer peak was also apparent, occurring in February of most years. A downward temporal trend was apparent, with some inter-annual variation around the trend.

Pearson correlations between overall incidence in the months January to December and the preceding 1–12 months are presented in additional file 1. Two clear patterns were apparent: firstly, there was strong temporal autocorrelation between incidence in each month and the preceding month; second, there were strong correlations between the months May–October and the preceding January and February. There was relatively little correlation between any month and a month in the preceding year, except that the summer peaks for *P. vivax *were correlated with the magnitude of the preceding summer peak.

**Figure 3:**
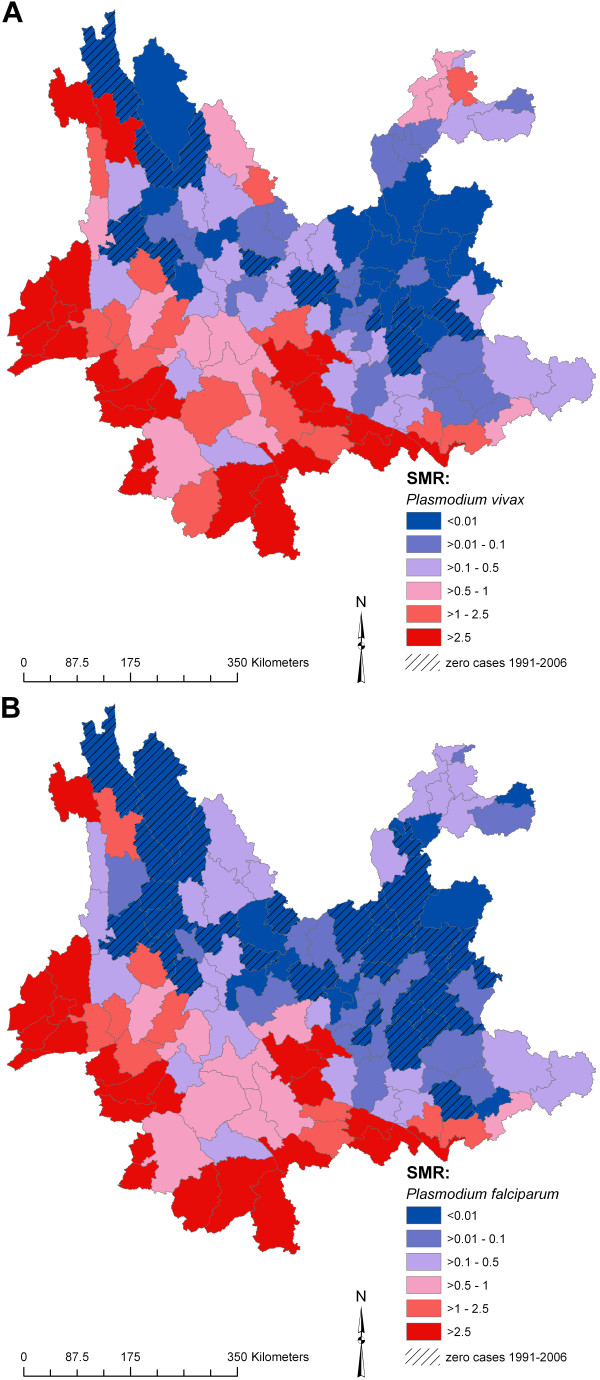
**Raw standardized morbidity ratios for incident cases of a) *Plasmodium vivax *and b) *P. falciparum *malaria in counties of Yunnan, China, 1991–2006**.

Maps of crude SMRs for the 128 counties of Yunnan for 1991–2006 (Figure 3) show a high incidence of both types of malaria among counties bordering Myanmar, Laos and Vietnam and counties located in the Red River (Hong He) valley. Low incidence counties were mainly located in the central region, around Kunming, the provincial capital, the eastern regions bordering Guizhou province and the north-western regions bordering Tibet.

### Spatio-temporal model

**Figure 4:**
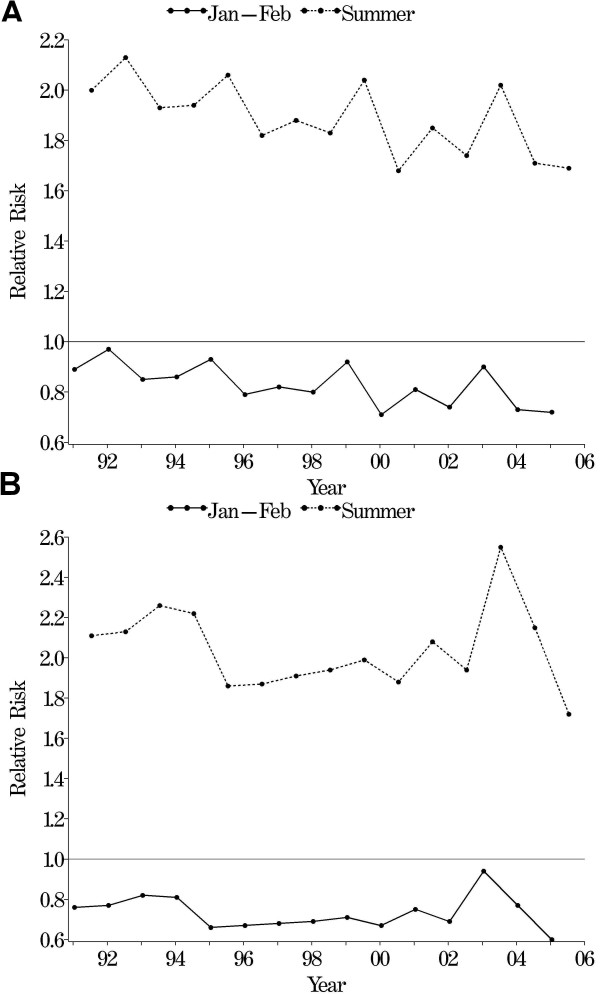
**Time series of modelled relative risks of a) *Plasmodium vivax *and b) *P. falciparum *malaria in January–February and June–September (summer), Yunnan, China, 1991–2006**.

The final spatio-temporal models are presented in Table 1. For both types of malaria, incidence in January–February was a significant predictor of incidence in the following June–September (as indicated by 95% CrI of the regression slope that excluded zero) meaning that higher than average incidence in January–February could predict higher than average incidence in the subsequent June–September period. Time series plots of the modelled relative risks in January–February and June–September highlighted the dependence of the summer incidence on the preceding January–February (Figure 4). Interestingly for *P. vivax*, the relative risks appeared to cycle every 3 to 4 years, with noticeably higher risks in 1992, 1995, 1999 and 2003, whereas for *P. falciparum *the pattern was less regular, with peaks in 1993–1994 and 2003. There were significant positive relationships between malaria incidence and both rainfall and maximum temperature (Table 1). There was a significant downward temporal trend in malaria incidence between 1991 and 2006, with an average decrease in incidence of 5.2% per year (95% CrI 4.8, 5.6%) for *P. vivax *and 4.3% per year (95% CrI 3.5, 5.1%) for *P. falciparum *across the province.

**Table 1:** Bayesian Poisson regression models of *Plasmodium vivax *and *P. falciparum *malaria, Yunnan, China, 1991–2006.

Variable	*Plasmodium vivax*	*Plasmodium falciparum*
*Relative Risks*		
Monthly rainfall (10 ml increase)	1.045 (1.044, 1.046)	1.037 (1.034, 1.040)
Monthly maximum temperature (°C increase)	1.047 (1.045, 1.050)	1.053 (1.047, 1.060)
Provincial average temporal trend (annual increase)	0.948 (0.944, 0.952)	0.957 (0.949, 0.965)
*Regression of June–September on January–February (log incidence)*		
Regression slope (Jan–Feb → Jun–Sep)	0.77 (0.70, 0.84)	0.90 (0.75, 1.09)
*Variance components (variances on a scale of log incidence)*		
Spatial random effect	8.74 (7.90, 9.89)	12.66 (10.50, 15.58)
Spatially-smoothed county-level temporal trend	0.08 (0.06, 0.10)	0.01 (0.00, 0.01)
Seasonal effect (January–February)	0.02 (0.01, 0.04)	0.02 (0.01, 0.06)
Overall Intercept	-2.52 (-2.60, -2.45)	-3.24 (-3.46, -3.04)

Results show mean and 95% credible interval (CrI). Summaries of the posterior distributions for the relative risks for each season are presented in the additional materials and the means are plotted in Figure 3.

**Figure 5:**
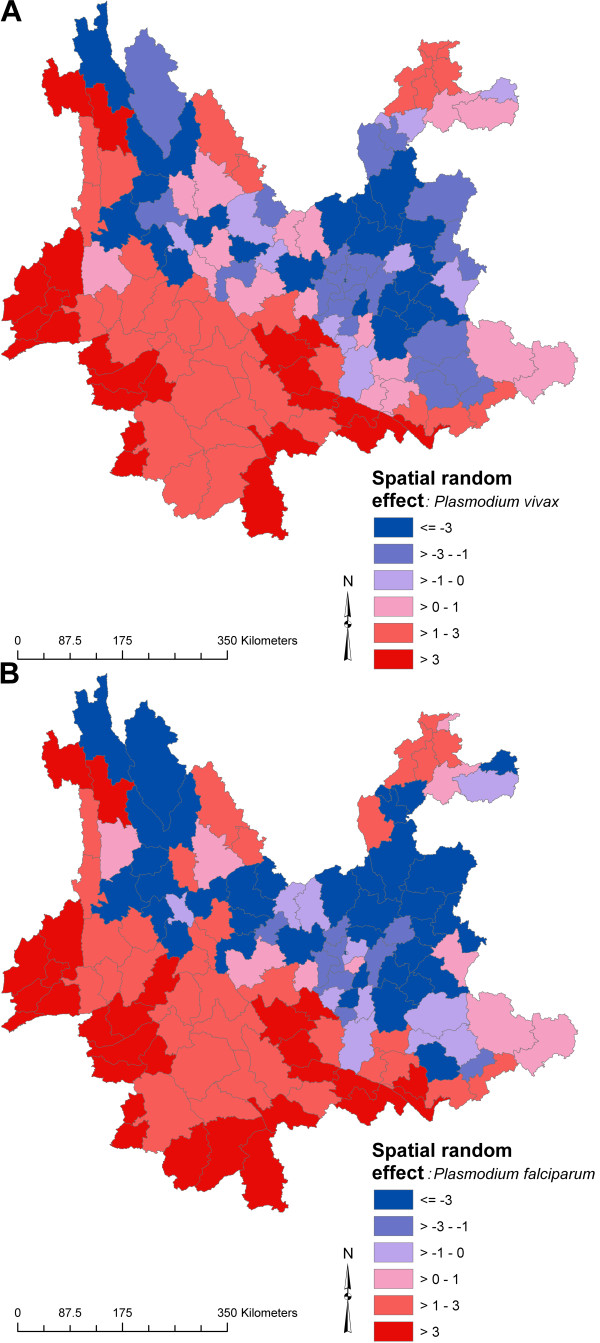
**Spatial random effects for a) *Plasmodium vivax *and b) *P. falciparum *malaria, Yunnan, China, 1991–2006**. Values are on a scale of log relative risk (values < 0 indicate lower than average risk, values > 0 indicate higher than average risk).

**Figure 6:**
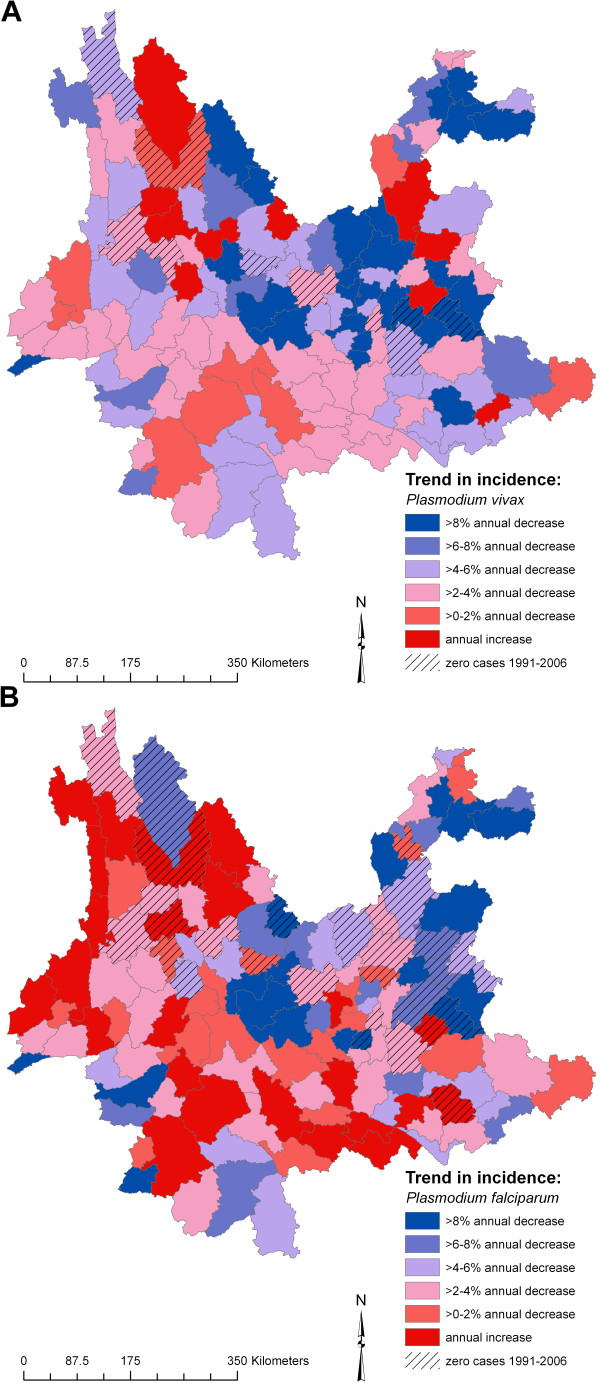
**Spatially smoothed temporal trend for a) *Plasmodium vivax *and b) *P. falciparum *malaria, Yunnan, China, 1991–2006**.

Maps of the spatial random effect, representing residual spatial clustering after accounting for the climate variables, are presented in Figure 5. For both types of malaria, clusters of high-incidence counties were located in south-western Yunnan and far northern Yunnan, whereas clusters of low-incidence counties were located in eastern and north-western Yunnan. A map of the spatial variation in county-level temporal trend is presented in Figure 6. Clusters of counties with higher than average temporal trend (i.e., a gentler decline in incidence over the 16 years) were interspersed with clusters of counties with lower than average temporal trend, throughout the province. Clusters of counties in north-western and north-eastern Yunnan actually showed an increasing trend in incidence for *P. vivax*, albeit with very low overall SMRs.

## Discussion

Characterizing spatial and temporal patterns of clinical malaria provides insights into the important drivers of this disease, including climatic variables such as rainfall and temperature that influence seasonal patterns, and human factors that influence long-term trends. Additionally, the model results suggest that the burden of malaria in the summer months can be predicted by the incidence of malaria in January and February. Incidence in January and February could, therefore, be used as a MEWS to prepare areas of Yunnan province for additional resource needs and emergency measures, leading to more timely and efficient responses to the anticipated disease burden in summer. Using monthly signals within a MEWS demands long-term, time-series data. How the temporal signals change with time as overall monthly incidence declines (as part of a systematic downward trend) requires further prospective investigation.

The association between incidence in summer and the preceding January–February might be explained by intrinsic host-pathogen dynamics whereby the number of cases in January–February determines the subsequent numbers of infected mosquitoes and infectious contacts between mosquitoes and susceptible people later in the season. Intra- and inter-annual variation, represented by short and long-term oscillations in malaria incidence, might also be explained by intrinsic host-pathogen dynamics independent of climate variation [25,26]. Climate-driven mathematical models of malaria transmission that incorporate intrinsic host-pathogen dynamics have been developed [27]. As an area of further research, models will be developed to investigate the influences of climate and host-pathogen dynamics on observed variation in malaria incidence in Yunnan. Such models can be extended to investigate and evaluate the impact of different interventions [28], providing a more comprehensive framework for planning malaria control.

The reasons for the January–February peak itself are not certain but it could be explained as the initial wave of annually recurring epidemics. The parasites could be maintained in the population during the cool, dry season by low-level transmission (or hibernation of *P. vivax *in the liver of infected people) or be introduced by immigration of infectious people from neighbouring regions. The first increases in rainfall and temperature in January–February could trigger increases in the mosquito population that result in the first wave of transmission, precipitating the next annual cycle. Further investigation is required to confirm this scenario but other factors such as patterns of reporting (especially around lunar New Year), migration and agricultural practices need to be considered.

As with other studies in Asia [8,29] and increasingly in parts of Africa [30,31], a downward trend in both *P. vivax *and *P. falciparum *malaria incidence in Yunnan was identified. This was is likely to be associated with general economic development, changing agricultural practices, increasing urbanisation and better access to healthcare. Trends in malaria incidence are also likely to be influenced by interventions. In addition to support from the ongoing national malaria control programme, Yunnan has been a recipient of three rounds (1, 5 and 6) of funding by the Global Fund to Fight AIDS, Tuberculosis and Malaria [32]. The Yunnan components of these projects have been managed by the Yunnan Institute of Parasitic Diseases. The aim has been to provide comprehensive preventive interventions (based on bed nets, anti-malarial drugs, vector control and health education) and to strengthen local health systems and surveillance capabilities. Round one (2003–2008) targeted the 26 international border counties of Yunnan and round five (2006–2011) is currently targeting an additional 22 counties in the south, southwest and far north of the province. In round six (2007–2009), Chinese migrant workers from 12 counties crossing the Myanmar border plus inhabitants of bordering areas of Myanmar are targeted. Other small projects in Yunnan have focussed on malaria in ethnic minority groups and include the Mekong Roll Back Malaria Information, Education and Communication project (2002–2004) and the Strengthening Malaria Control for Ethnic Minorities project (2006–2007), supported by the Asia Development Bank. The results presented in this report suggest that the downward trend in malaria incidence has preceded all of these internationally-funded malaria initiatives in the province.

The model outputs demonstrated that reported malaria cases predominantly occur in spatial clusters of counties. Where spatial clustering is an intrinsic feature of infectious disease data, it is critical to accommodate this phenomenon in statistical analyses to avoid violation of the assumption that observations are independent. Furthermore, the identification of clusters can provide a public health tool to investigate reasons for spatial clustering. Clusters located adjacent the international borders were probably partly due to the previously described cross-border migration of people from areas of poor malaria control. While not formally tested, there is a striking congruence between the spatial random effect map (Figure 5) and Ahmad and Goh's [33] poverty map of Yunnan, suggesting a role of socioeconomic factors in the distribution of malaria. Other factors that might play a role in clustering of disease events over variable spatial scales (but which could not be explored in this study) include location of communities in relation to local vector breeding sites, intrinsic heterogeneity due to disease dynamics and pockets of antimalarial drug resistance.

It is acknowledged that there were likely to have been imperfections in the data given that they were obtained from a passive surveillance system. According to a 2005 national report, it was estimated that only 1/18 (5.6%) cases in China are notified [34] and variations in reporting by county might have impacted on observed spatial patterns. Malaria diagnosis was also imperfect, with possible confusion between malaria due to *P. vivax *and *P. falciparum *and other diseases causing similar clinical signs. It is currently unknown to what extent the case definitions used, or the proportion of cases confirmed by microscopy, have varied between locations or through time.

Robust Bayesian statistical methods were employed to quantify seasonal and sub-provincial variation of *P. vivax *and *P. falciparum *malaria and the effects of climatic factors. This work indicated the important roles of rainfall and temperature in driving spatio-temporal patterns of malaria incidence in Yunnan and provided evidence that intrinsic host-pathogen dynamics might also contribute, setting the scene for future mathematical modelling work. While the provincial average long-term trend of malaria incidence indicates general success in reducing the malaria burden (through direct or indirect means), this success has not been evenly distributed throughout Yunnan (Figure 6), and spatial heterogeneities remain (Figure 5). Targeted distribution of resources should be implemented using evidence-based approaches, augmented by spatio-temporal analytical methods, to assist more effective malaria control in counties of Yunnan where these resources are most needed.

## Competing interests

The authors declare that they have no competing interests.

## Authors' contributions

ACAC conducted the analysis and drafted the manuscript, AGB assisted with the statistical analysis and created figures 2 and 4, ZWC collated the surveillance and climatic datasets and RWS and HNZ provided extensive comments on the manuscript. All authors read and approved the final manuscript.

## Supplementary Material

Additional file 1 **Additional statistical analysis materials**. The file contains tables showing the cross-correlations between raw malaria incidence in each month and the preceding months and the modelled values of log relative risk for each of the January–February and June–September periods from 1991–2006. The file also contains figures showing the median long-term average monthly rainfall and temperature by Yunnan county. Click here for file

Additional file 2 **Bayesian spatial model**. The file contains statistical notation of the Bayesian spatial models used in the manuscript. Click here for file
