# Supplementary material for: Space-time variation of malaria incidence in Yunnan province, China
Source: Malar J. 2009 Jul 31;8:180. doi: 10.1186/1475-2875-8-180 (PMC2724544; doi:10.1186/1475-2875-8-180)
Supplement: Additional file 1 — Additional statistical analysis materials. The file contains tables showing the cross-correlations between raw malaria incidence in each month and the preceding months and the modelled values of log relative risk for each of the January–February and June–September periods from 1991–2006. The file also contains figures showing the median long-term average monthly rainfall and temperature by Yunnan county. [file 1475-2875-8-180-S1.doc]

Additional materials

Table 1. Pearson cross-correlations between the observed number of cases of a) *Plasmodium vivax* and b) *P. falciparum* malaria in each month and previous months. Calculations were made for a lag of 1–12 months; the diagonal in the table (shaded grey) corresponds to the same month in the preceding year, values above the diagonal indicate correlations with preceding months in the same year and values below the diagonal indicate correlations with preceding months in the preceding year. Correlation coefficients above 0.8 are in bold.

1. *P. vivax* malaria

| Preceding Month | Month | | | | | | | | | | | |
| --- | --- | --- | --- | --- | --- | --- | --- | --- | --- | --- | --- | --- |
|  | Jan | Feb | Mar | Apr | May | Jun | Jul | Aug | Sept | Oct | Nov | Dec |
| Jan | 0.65 | 0.75 | **0.93** | **0.91** | **0.89** | **0.94** | **0.88** | **0.86** | **0.94** | **0.91** | 0.70 | **0.84** |
| Feb | 0.70 | 0.73 | 0.78 | 0.61 | **0.92** | **0.84** | **0.96** | **0.97** | **0.92** | 0.67 | 0.35 | 0.46 |
| Mar | 0.57 | **0.80** | 0.65 | **0.96** | **0.95** | **0.99** | **0.90** | **0.83** | **0.92** | **0.96** | 0.78 | **0.80** |
| Apr | 0.50 | 0.72 | 0.58 | 0.45 | **0.85** | **0.93** | 0.78 | 0.69 | **0.83** | **0.96** | **0.86** | **0.90** |
| May | 0.71 | **0.81** | 0.77 | 0.65 | **0.85** | **0.98** | **0.98** | **0.94** | **0.96** | **0.87** | 0.61 | 0.64 |
| Jun | 0.67 | **0.82** | 0.74 | 0.61 | **0.82** | 0.78 | **0.94** | **0.88** | **0.95** | **0.94** | 0.73 | 0.76 |
| Jul | 0.72 | **0.84** | 0.77 | 0.65 | **0.87** | **0.82** | **0.86** | **0.98** | **0.99** | **0.82** | 0.53 | 0.62 |
| Aug | 0.75 | **0.84** | 0.77 | 0.66 | **0.87** | **0.82** | **0.86** | **0.86** | **0.97** | 0.75 | 0.43 | 0.57 |
| Sept | 0.74 | **0.88** | 0.77 | 0.65 | **0.88** | **0.82** | **0.89** | **0.89** | **0.87** | **0.86** | 0.58 | 0.71 |
| Oct | 0.52 | 0.74 | 0.59 | 0.46 | 0.66 | 0.61 | 0.71 | 0.72 | 0.69 | 0.44 | **0.89** | **0.88** |
| Nov | 0.20 | 0.43 | 0.25 | 0.18 | 0.29 | 0.25 | 0.37 | 0.39 | 0.37 | 0.16 | -0.02 | **0.84** |
| Dec | 0.30 | 0.62 | 0.33 | 0.24 | 0.43 | 0.36 | 0.52 | 0.57 | 0.51 | 0.18 | -0.01 | 0.17 |

1. *P. falciparum* malaria

| Preceding Month | Month | | | | | | | | | | | |
| --- | --- | --- | --- | --- | --- | --- | --- | --- | --- | --- | --- | --- |
|  | Jan | Feb | Mar | Apr | May | Jun | Jul | Aug | Sept | Oct | Nov | Dec |
| Jan | 0.66 | **0.90** | 0.73 | 0.60 | **0.83** | **0.93** | **0.96** | **0.98** | **0.93** | **0.82** | 0.34 | 0.49 |
| Feb | 0.68 | 0.74 | **0.93** | 0.78 | **0.90** | **0.97** | **0.98** | **0.95** | **0.98** | **0.96** | 0.64 | 0.76 |
| Mar | 0.70 | 0.70 | 0.63 | **0.93** | **0.90** | **0.88** | **0.85** | 0.79 | **0.92** | **0.93** | **0.81** | **0.94** |
| Apr | 0.64 | 0.62 | 0.55 | 0.48 | **0.88** | 0.78 | 0.71 | 0.63 | **0.82** | **0.85** | **0.86** | **0.96** |
| May | 0.64 | 0.60 | 0.50 | 0.44 | 0.47 | **0.93** | **0.88** | **0.85** | **0.93** | **0.91** | 0.69 | 0.79 |
| Jun | 0.68 | 0.72 | 0.68 | 0.60 | 0.55 | 0.69 | **0.98** | **0.96** | **0.97** | **0.97** | 0.63 | 0.71 |
| Jul | 0.70 | 0.76 | 0.71 | 0.63 | 0.57 | 0.71 | 0.76 | **0.98** | **0.98** | **0.92** | 0.52 | 0.64 |
| Aug | 0.71 | 0.76 | 0.70 | 0.60 | 0.56 | 0.71 | 0.77 | 0.74 | **0.96** | **0.87** | 0.41 | 0.56 |
| Sept | 0.75 | 0.78 | 0.72 | 0.64 | 0.61 | 0.75 | **0.80** | 0.77 | **0.80** | **0.94** | 0.61 | 0.76 |
| Oct | 0.64 | 0.66 | 0.62 | 0.53 | 0.49 | 0.63 | 0.69 | 0.67 | 0.69 | 0.62 | **0.80** | **0.82** |
| Nov | 0.35 | 0.34 | 0.32 | 0.26 | 0.27 | 0.35 | 0.38 | 0.37 | 0.37 | 0.32 | 0.15 | **0.90** |
| Dec | 0.58 | 0.57 | 0.51 | 0.43 | 0.48 | 0.57 | 0.60 | 0.57 | 0.59 | 0.55 | 0.32 | 0.42 |

Table 2. Relative risks for each season, derived from Bayesian Poisson regression models of *Plasmodium vivax* and *P. falciparum* malaria, Yunnan, China, 1991–2005. Results show posterior mean and 95% posterior interval. The means are plotted in Figure 5 of the main paper.

| **Season** | ***P. vivax*** | ***P. falciparum*** |
| --- | --- | --- |
| 1991, January–February | –0.12 (–0.20, –0.04) | –0.27 (–0.42, –0.11) |
| 1991, June–September | 0.69 (0.63, 0.76) | 0.75 (0.60, 0.90) |
| 1992, January–February | –0.03 (–0.11, 0.05) | –0.27 (–0.41, –0.11) |
| 1992, June–September | 0.76 (0.69, 0.82) | 0.75 (0.61, 0.90) |
| 1993, January–February | –0.16 (–0.23, –0.08) | –0.20 (–0.35, –0.04) |
| 1993, June–September | 0.66 (0.60, 0.72) | 0.81 (0.66, 0.97) |
| 1994, January–February | –0.15 (–0.22, –0.08) | –0.22 (–0.37, –0.06) |
| 1994, June–September | 0.66 (0.61, 0.72) | 0.80 (0.65, 0.95) |
| 1995, January–February | –0.08 (–0.15, 0.00) | –0.41 (–0.58, –0.26) |
| 1995, June–September | 0.72 (0.66, 0.78) | 0.62 (0.46, 0.77) |
| 1996, January–February | –0.24 (–0.31, –0.16) | –0.41 (–0.57, –0.25) |
| 1996, June–September | 0.60 (0.54, 0.66) | 0.63 (0.47, 0.78) |
| 1997, January–February | –0.20 (–0.27, –0.13) | –0.39 (–0.55, –0.23) |
| 1997, June–September | 0.63 (0.57, 0.69) | 0.64 (0.49, 0.79) |
| 1998, January–February | –0.23 (–0.30, –0.15) | –0.37(–0.53, –0.21) |
| 1998, June–September | 0.60 (0.55, 0.66) | 0.66 (0.51, 0.81) |
| 1999, January–February | –0.09 (–0.16, –0.02) | –0.34 (–0.50, –0.180) |
| 1999, June–September | 0.71 (0.66, 0.77) | 0.69 (0.54, 0.83) |
| 2000, January–February | –0.34 (–0.42, –0.26) | –0.40 (–0.57, –0.24) |
| 2000, June–September | 0.52 (0.45, 0.58) | 0.63 (0.47, 0.78) |
| 2001, January–February | –0.22 (–0.29, –0.14) | –0.29 (–0.45, –0.13) |
| 2001, June–September | 0.61 (0.55, 0.67) | 0.73 (0.58, 0.88) |
| 2002, January–February | –0.30 (–0.38, –0.22) | –0.37 (–0.53, –0.20) |
| 2002, June–September | 0.55 (0.48, 0.61) | 0.66 (0.51, 0.81) |
| 2003, January–February | –0.10 (–0.18, –0.03) | –0.06 (–0.24, 0.11) |
| 2003, June–September | 0.70 (0.64, 0.76) | 0.94 (0.77, 1.10) |
| 2004, January–February | –0.31 (–0.40, –0.23) | –0.26 (–0.42, –0.09) |
| 2004, June–September | 0.54 (0.47, 0.61) | 0.76 (0.61, 0.92) |
| 2005, January–February | –0.33 (–0.42, –0.25) | –0.50 (–0.71, –0.32) |
| 2005, June–September | 0.52 (0.45, 0.590 | 0.54 (0.35, 0.70) |
| 2006, January–February | –0.45 (–0.55, –0.35) | –0.47 (–0.67, –0.29) |
| 2006, June–September | 0.43 (0.35, 0.51) | 0.57 (0.39, 0.73) |


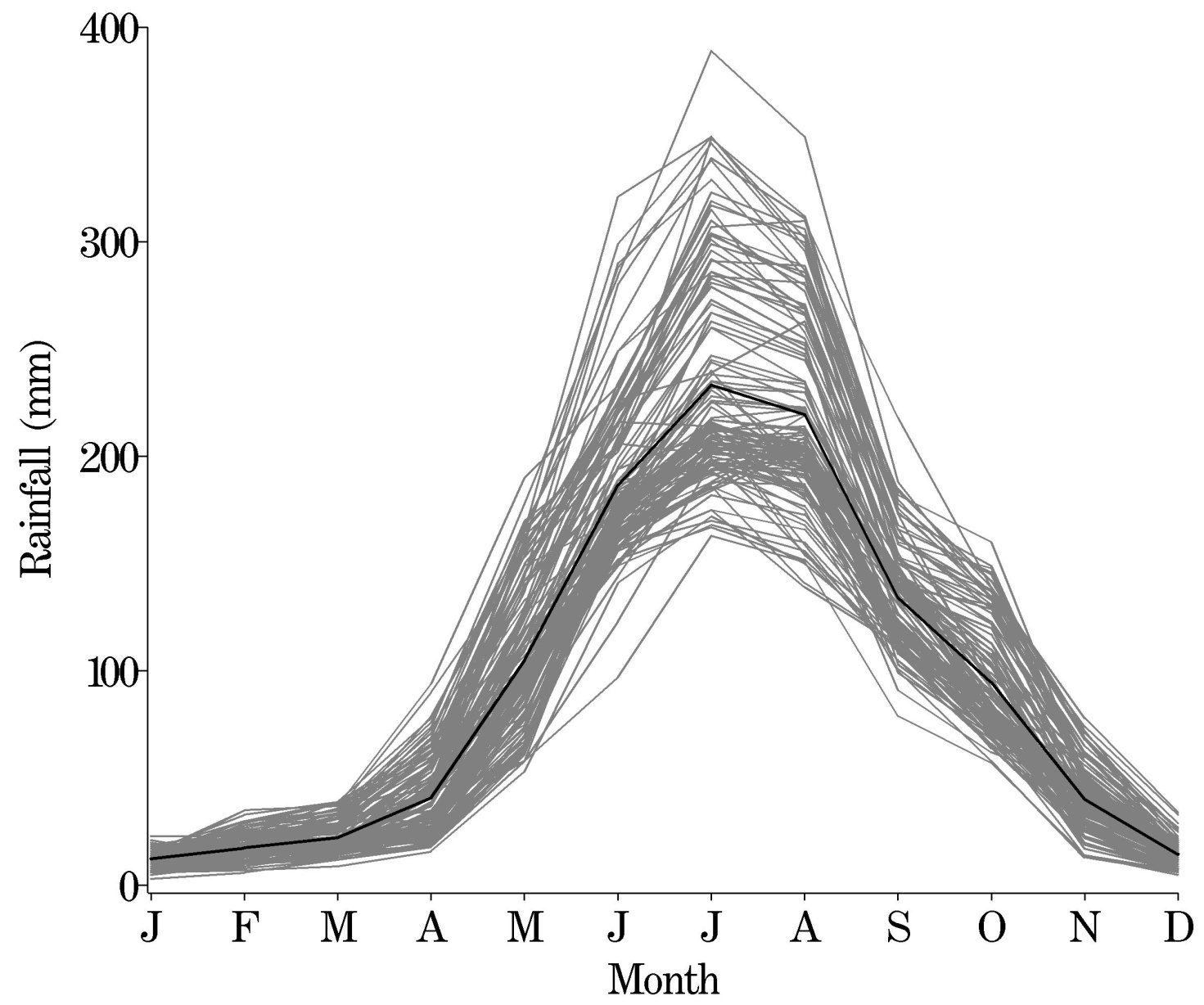


Figure 1. Median long-term average rainfall by county in Yunnan province, China. The provincial average is in black.


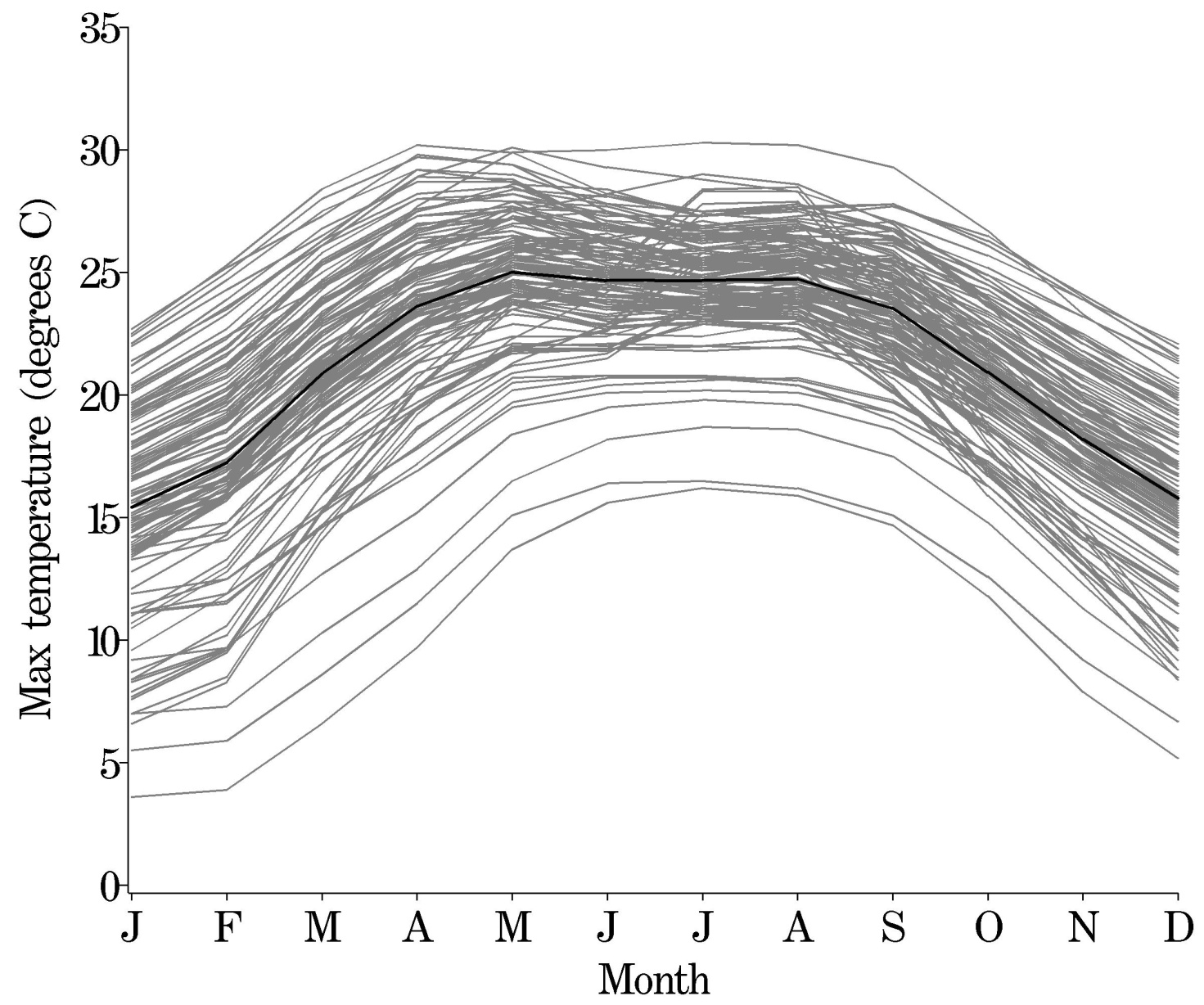


Figure 2. Median long-term average maximum temperature by county in Yunnan province, China. The provincial average is in black.
