# Supplementary material for: Space-time variation of malaria incidence in Yunnan province, China
Source: Malar J. 2009 Jul 31;8:180. doi: 10.1186/1475-2875-8-180 (PMC2724544; doi:10.1186/1475-2875-8-180)
Supplement: Additional file 2 — Bayesian spatial model. The file contains statistical notation of the Bayesian spatial models used in the manuscript. [file 1475-2875-8-180-S2.doc]

**Bayesian spatial model**

The final regression model was:

$O_{ij}\sim Poisson(Y_{ij})$,

where *O_ij_* is the observed number of cases in county *i*, month *j* and

$\log(Y_{ij})=\log(E_{ij})+\theta_{ijk}$,

where *E_ij_* (the expected number of cases in county *i*, month *j*) is an offset to control for population size. The mean log relative risk was modelled as:

$\theta_{ijk}=\alpha+\beta\times rain_{ij}+\delta\times temp_{ij}+\nu\times month_{j}+\varepsilon_{i}\times month_{ij}+s_{i}+\varphi_{jk}$,

where *α* is the intercept, *β* is the coefficient for rainfall, *δ* is the coefficient for maximum temperature, *ν* is the provincial average temporal trend coefficient, *ε_i_* are the spatially smoothed county-level temporal trend coefficients, *s_i_* is a spatial county-level random effect and

$\varphi_{jk}=\gamma_{1,k}\times I(month_{j}\in Jan-Feb)+\gamma_{2,k}\times I(month_{j}\in June-Sept)$,

where $\gamma_{1,k}$ represent incidence in January–February in year *k* and $\gamma_{2,k}$ represent incidence in June–September in year *k*. *I*(.) is an indicator function which is equal to one if the condition in the brackets is true and zero otherwise. We modelled the dependence between these two effects using a linear regression:

$\gamma_{2,k}=\lambda+\eta\times\gamma_{1,k}$,

where *λ* is the intercept for the regression of the June–September on the preceding January–February, and *η* is its slope. We used the cut function on $\gamma_{1,k}$ to ensure that the dependence in incidence rates went from Jan–Feb to June–Sept, and did not flow the other way.

Spatial structuring in *s_i_* and *ε_i_* was modelled using a conditional autoregressive prior structure, where spatial relationships between counties were modelled using a simple adjacency weights matrix. If two counties were adjacent, the weight = 1 and if they were not adjacent, the weight = 0.
